# An adjuvanted subunit SARS-CoV-2 spike protein vaccine provides protection against Covid-19 infection and transmission

**DOI:** 10.1038/s41541-022-00450-8

**Published:** 2022-02-23

**Authors:** Kairat Tabynov, Nurkeldi Turebekov, Meruert Babayeva, Gleb Fomin, Toktassyn Yerubayev, Tlektes Yespolov, Lei Li, Gourapura J. Renukaradhya, Nikolai Petrovsky, Kaissar Tabynov

**Affiliations:** 1 International Center for Vaccinology, Kazakh National Agrarian Research University (KazNARU), Almaty, Kazakhstan; 2 Preclinical Research Laboratory with Vivarium, M. Aikimbayev National Research Center for Especially Dangerous Infections, Almaty, Kazakhstan; 3 Central Reference Laboratory, M. Aikimbayev National Scientific Center for Especially Dangerous Infections, Almaty, Kazakhstan; 4 Vaxine Pty Ltd and Flinders University, Bedford Park, Australia; 5 Center for Food Animal Health, Ohio Agricultural Research and Development Center, The Ohio State University (OSU), Wooster, OH 44691 United States

**Keywords:** Protein vaccines, Viral infection

## Abstract

Recombinant protein approaches offer major promise for safe and effective vaccine prevention of SARS-CoV-2 infection. We developed a recombinant spike protein vaccine (called NARUVAX-C19) and characterized its ability when formulated with a nanoemulsion adjuvant to induce anti-spike antibody and T-cell responses and provide protection including against viral transmission in rodent. In mice, NARUVAX-C19 vaccine administered intramuscularly twice at 21-day interval elicited balanced Th1/Th2 humoral and T-cell responses with high titers of neutralizing antibodies against wild-type (D614G) and delta (B.1.617.2) variants. In Syrian hamsters, NARUVAX-C19 provided complete protection against wild-type (D614G) infection and prevented its transmission to naïve animals (*n* = 2/group) placed in the same cage as challenged animals (*n* = 6/group). The results contrasted with only weak protection seen with a monomeric spike receptor-binding domain (RBD) vaccine even when formulated with the same adjuvant. These encouraging results warrant the ongoing development of this COVID-19 vaccine candidate.

## Introduction

Currently, the world remains in the pandemic caused by Severe Acute Respiratory Syndrome Coronavirus - 2 (SARS-CoV-2). This pandemic has affected every country in the world, and as of Nov 2021, confirmed cases totaled over 253 million, with at least 5.1 million deaths^1^. New variants continue to emerge, making containment strategies difficult^1^, due to immune escape^2,3^. Countries with high vaccination coverage have seen reduced hospitalizations and deaths as a percentage of infection cases^1^, suggesting vaccines may reduce the severity and health impacts of COVID-19 infection. Much weaker evidence is available on the role of current vaccines in reducing virus transmission in the community, with high rates of virus spread witnessed with the relaxation of social isolation policies even in countries with high vaccination coverage^4^.

Several vaccines (inactivated-, mRNA-, and vector-based) have emergency authorization^5^, but remain insufficient to meet global demand. So far with over 4 billion doses of vaccine distributed, just 14.9% of the world’s population has been fully vaccinated, with only 1.1% of people in low-income countries having received at least one dose^6^. The development of additional vaccines with different mechanisms of action could help in mitigating the global impact of SARS-CoV-2, with a major missing link being the ongoing lack of availability of traditional recombinant protein subunit vaccines. Protein-based vaccines have advantages over other technologies with a 40-year record of high safety and efficacy including in young infants, plus the low cost of goods, reliable large-scale production and stability under normal refrigeration conditions^7^.

We developed a subunit COVID-19 vaccine called NARUVAX-C19 based on recombinant spike (rSpike) protein extracellular domain (ECD) expressed in insect cells that was then formulated with Sepivac SWE™, a nanoemulsion oil (SWE) adjuvant. In this study, we evaluated the antibody and T-cell responses and protective efficacy of the NARUVAX-C19 vaccine including against virus transmission. For comparison, vaccines based on recombinant monomeric receptor-binding domain (rRBD) protein formulated with oil adjuvants were also included. This is one of the first studies to directly compare spike ECD protein with monomeric RBD antigens to evaluate protection against SARS-CoV-2 infection and the ability of such vaccines to block viral transmission. The study showed that NARUVAX-C19 vaccine-induced potent humoral and cellular immune responses in mice and protected hamsters against viral challenge with the prevention of virus transmission to naïve animals

## Results

### Antibody response in vaccinated mice

**Fig. 1:**
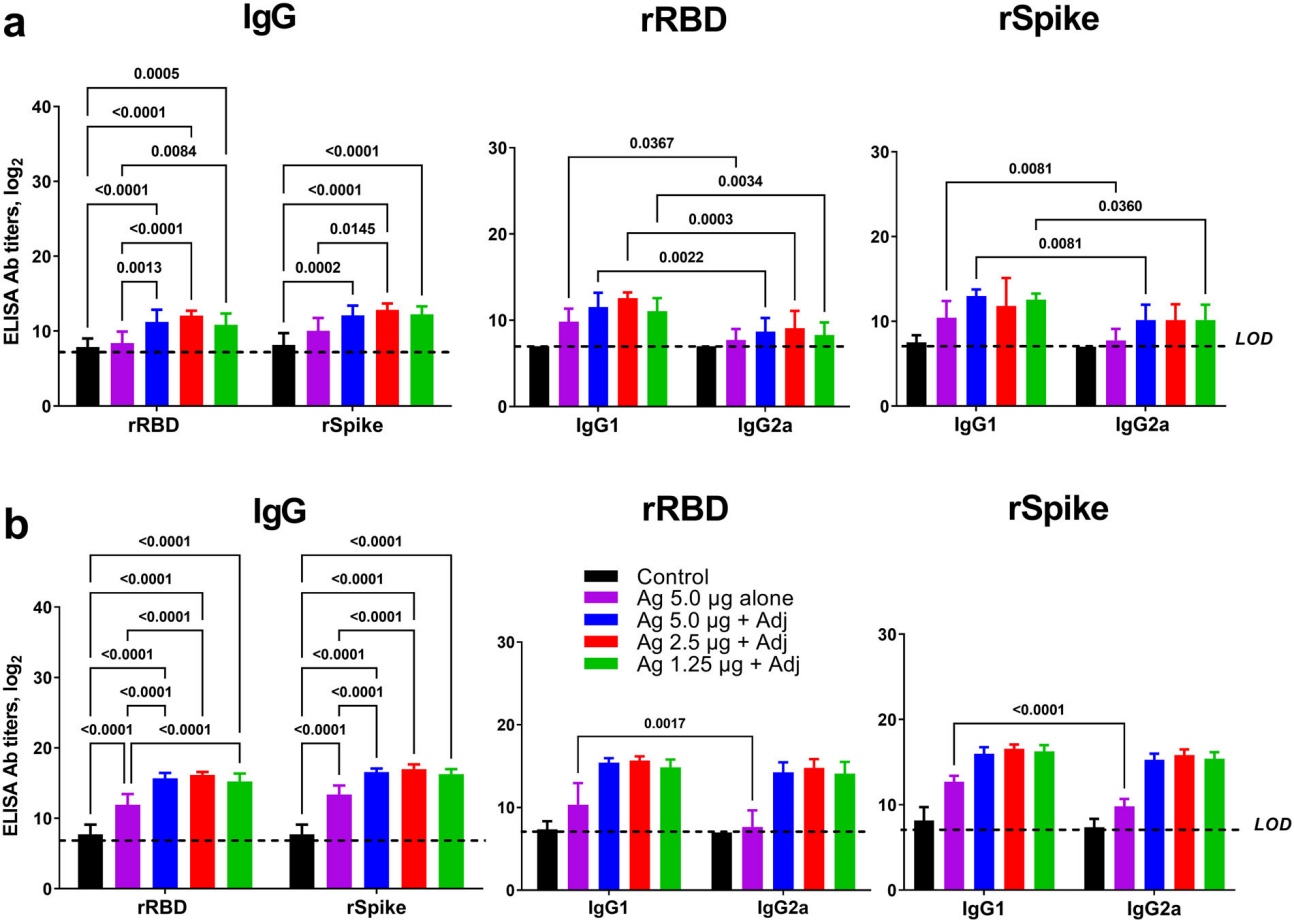
Antigen-specific IgG, IgG1 and IgG2a titers in serum of BALB/c mice vaccinated with rRBD or rSpike protein ECD at 21 days after prime and booster immunization. **a** Data post prime vaccination, **b** data post booster vaccination. Antigens were used at doses of 1.25, 2.5, and 5 µg with SWE adjuvant. For comparison, 5 µg vaccine was given without adjuvant and a negative control vaccine comprised SWE adjuvant with no antigen. Antibody levels are presented as geometric mean titers (GMT) with a 95% confidence interval. Differences in IgG titers were assessed using Tukey’s multiple comparisons test. A *P* < 0.05 value was considered as a significant difference.

In mice, both rSpike and rRBD proteins formulated with SWE adjuvant-induced serum spike-specific IgG 21 days after a single immunization (Fig. 1a). Spike-specific IgG levels were further boosted after the second immunization (Fig. 1b), with significantly higher IgG in groups that received adjuvanted versus unadjuvanted antigens. Notably, for unadjuvanted antigen anti-spike or RBD IgG was only seen after two doses. Both IgG1 and IgG2 subtypes were produced against spike and RBD protein. At 21 days after booster vaccination (Fig. 1b), IgG1 and IgG2a antibody titers in all the adjuvanted vaccinated groups was equivalent indicating a balanced Th1 and Th2 response. By contrast, the unadjuvated antigens induced predominantly IgG1 consistent with a Th2 biased response.

**Fig. 2:**
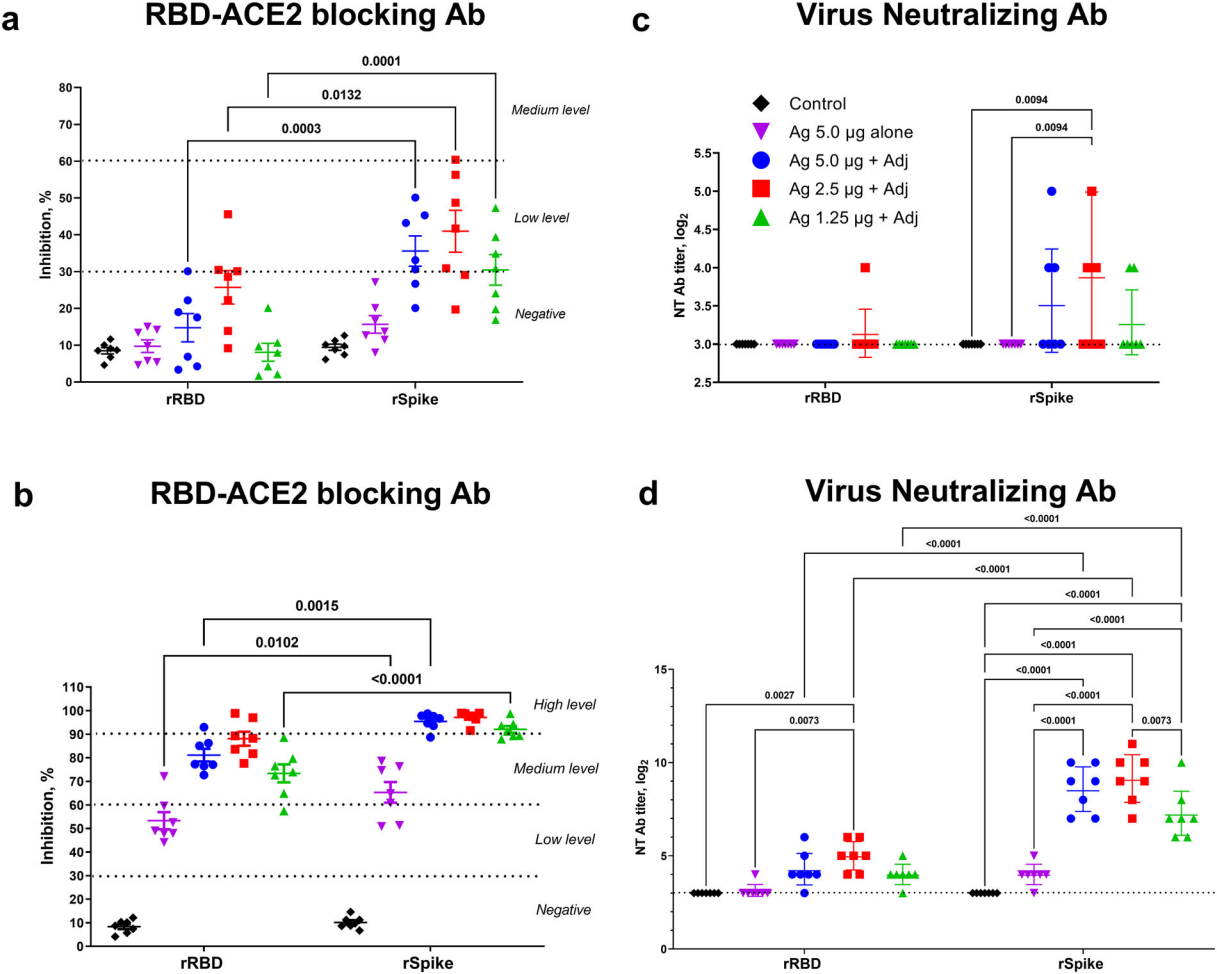
RBD-ACE2 blocking and virus-neutralizing antibody titers in BALB/c mice at 21 days after prime and booster immunization with SWE-adjuvanted rRBD and rSpike protein vaccines. **a**, **c** Data post prime vaccination, **b**, **d** data post booster vaccination. Controls included rRBD or rSpike protein alone (Ag 5.0 µg alone) or SWE adjuvant alone (Control). RBD-ACE2 blocking antibodies were determined according to the level of inhibition: negative (<30%), low (30–59%), medium (60–89), and high (90≤). Virus neutralizing antibody levels are presented as geometric mean titers (GMT) with a 95% confidence interval. Differences in antibody titers between groups were assessed using Tukey’s multiple comparisons test.

We next determined the ability of immune sera to block the binding of SARS-CoV-2 RBD protein to the ACE2 receptor. The SWE-adjuvanted rSpike protein vaccine generated RBD-ACE2 blocking antibodies in 57.1–71.4% of mice at tested doses as early as 21 days after a single immunization, which was significantly higher than that of the rRBD-based vaccine (0–42.8%) (Fig. 2a). After a single dose, RBD-ACE2 blocking antibodies were not detected in animals immunized with unadjuvanted rRBD or rSpike antigens alone. At 21 days after the booster immunization, the percentage of mice with RBD-ACE2 blocking antibodies in all vaccinated groups reached 100% (Fig. 2b). Overall, the rSpike protein-immunized groups demonstrated higher RBD-ACE2 blocking antibody levels that the rRBD-immunized groups.

At 21 days after prime immunization, neutralizing antibodies against the wild-type SARS-CoV-2 virus were detected in at least some animals in each of the SWE-adjuvanted rSpike protein groups (Fig. 2c) but just one animal in just one of the SWE-adjuvanted rRBD (2.5 µg) groups. After booster immunization (Fig. 2d), the adjuvanted rSpike protein groups at all three doses had high neutralizing antibodies titers (geometric mean titers [GMT] 475-706), which were significantly higher than the antigen alone group (GMT 11-20), the controls, and the corresponding groups immunized with SWE-adjuvanted rRBD antigen (GMT 20-40).

### T-cell immune response in vaccinated mice

**Fig. 3:**
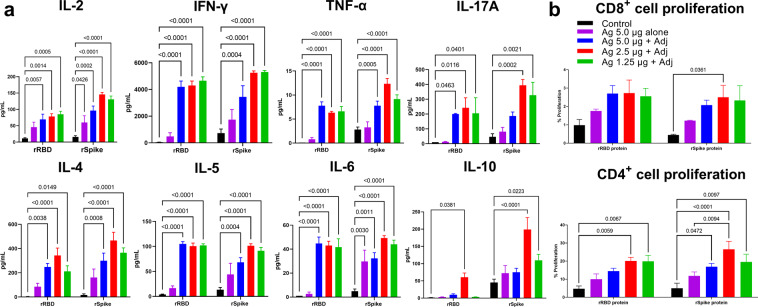
Antigen-stimulated cytokine production and CD4 + and CD8 + T cell proliferation in splenocytes from rRBD or rSpike protein vaccinated BALB/c mice. Controls included rRBD or rSpike protein alone at 5.0 µg (Ag 5.0 µg alone) and SWE adjuvant-alone group (Control). Cytokine data (**a**) were presented as the difference (delta) in cytokine concentrations between samples with and without protein stimulation. CD4 + and CD8 + T cell proliferation (**b**) was calculated as the difference (Δ) in number of proliferating (CFSE + ) lymphocytes between stimulated vs non-stimulated cells. Statistical differences were assessed using Šídák’s multiple comparisons test. A *P* < 0.05 value was considered as a significant difference.

After two immunizations, both the SWE-adjuvanted rRBD and rSpike protein-induced robust cellular immune responses (Fig. 3a). SWE-adjuvanted rRBD and rSpike protein-induced significantly higher production of both Th1 (IFN-gamma, IL-2, TNF-α, IL-17A) and Th2 (IL-4, IL-5, IL-6, IL-9, IL-10) cytokines compared to antigen-alone and SWE-alone control groups. Production of IL-2, TNF- α, and IL-10 were higher in the SWE-adjuvanted rSpike protein group (2.5 µg) compared to the SWE-adjuvanted rRBD vaccine. Hence, two doses of SWE-adjuvanted rSpike or rRBD protein-induced both Th1 and Th2 memory T cell responses.

Similar patterns were observed in antigen-stimulated CD4 + and CD8 + T cell proliferation responses (Fig. 3b). Again, SWE-adjuvanted rRBD and rSpike protein-induced significantly higher CD4 + T cell proliferation the controls when stimulated with the corresponding rRBD or rSpike proteins. The CD8 + cell proliferation for SWE-adjuvanted rRBD and rSpike protein trended higher than the control groups, but apart from the SWE-adjuvanted rSpike protein 2.5 µg group, these differences did not achieve statistical significance.

### Vaccine immunogenicity in hamsters

The immunogenicity of our vaccine candidates in hamsters were evaluated at Day 21 after a second intramuscular immunization. A single antigen dose of 5.0 µg/animal was used and no antigen-only groups were included, as in the mouse studies antigen alone did not induce neutralizing antibodies or a strong T cell response. Responses in vaccinated animals were compared to serum samples collected 21 days post-infection of unvaccinated hamsters with wild-type D614G SARS-CoV-2 virus (WT convalescent sera).

**Fig. 4:**
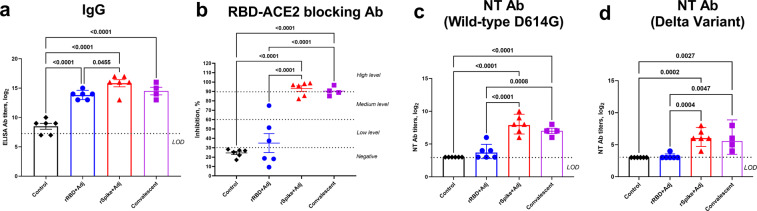
Spike-specific IgG, RBD-ACE2 blocking antibody and neutralizing antibody levels in hamsters at 21 days after booster immunization with SWE-adjuvanted rRBD or rSpike protein as compared to convalescent sera. **a** Data for spike-specific IgG, **b** RBD-ACE2 blocking antibody, **c**, **d** neutralizing antibody levels. Control refers to a group of animals injected with adjuvant-alone with no antigen. Convalescent refers to post-infection serum samples from four unvaccinated hamsters that had previously been challenged with the wild-type SARS-CoV-2 virus. Viral neutralizing antibodies were assessed against wild-type D614G (**c**) and delta variant (**d**) viruses. Differences between groups assessed using Dunnett’s multiple comparisons test.

In the hamsters, SWE-adjuvanted rSpike protein-induced significantly higher anti-spike IgG titers (GMT 35,918) than SWE-adjuvanted rRBD vaccine (GMT 14,254) vaccine (Fig. 4a) with the anti-spike IgG levels in hamsters immunized with SWE-adjuvanted rSpike protein equalling or exceeding levels seen in WT convalescent sera.

Hamsters immunized with SWE-adjuvanted rSpike protein had either high (4/6 animals) or medium (2/6 animals) levels of RBD-ACE2 blocking antibodies, equivalent to WT convalescent sera (Fig. 4b). By contrast, SWE-adjuvanted rRBD vaccine-induced RBD-ACE2 blocking antibody at low to medium levels in only 50% of animals.

Next, sera were assessed for neutralizing activity against wild-type D614G or the delta variant of SARS-CoV-2 virus. Consistent with the high levels of specific IgG and RBD-ACE2 blocking antibodies in the SWE-adjuvanted rSpike protein group, this group also had the highest titers of neutralizing antibodies (GMT 320), higher than levels in WT convalescent sera (GMT 160) and significantly higher than levels in the SWE-adjuvanted rRBD group (GMT 17.8, Fig. 4c).

Cross-neutralizing responses against the SARS-CoV-2 delta variant were detectable in 100% of animals that received SWE-adjuvanted rSpike (GMT 89.8*)* with levels higher than the WT convalescent sera (GMT 67.2), whereas they were undetectable in the majority of animals that received SWE-adjuvanted rRBD. Overall, neutralizing activity against the delta variant was ~threefold lower than against the wild-type virus in the vaccinated animals with a similar trend apparent in WT convalescent sera (Fig. 4d).

### Vaccine efficacy and transmission studies in hamsters

**Fig. 5:**
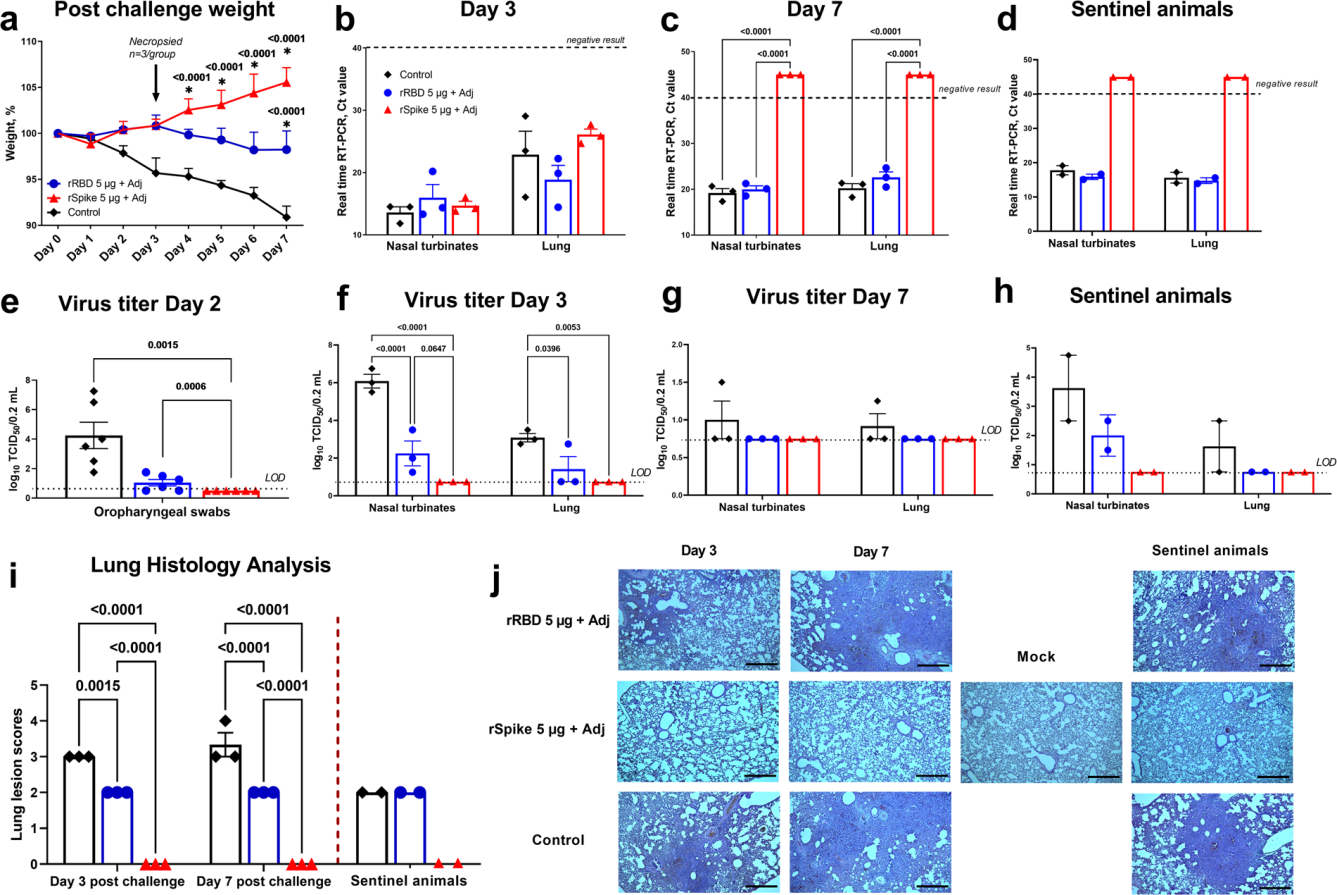
Vaccine protection in Syrian hamsters against SARS-CoV-2 infection and virus transmission. To assess vaccine efficacy animals (*n* = 6/group) were intranasally infected with WT SARS-CoV-2 and the following parameters then measured: 1) changes in body weight (**a**; *n* = 3-6/group); virus excretion from oropharyngeal swabs Day 2 post challenge (**e**; *n* = 6/group) 2) viral load in nasal turbinates (*n* = 3/group) and lungs (*n* = 3/group) by PCR (represented by Ct; **b**, **c**) and culture of infectious virus (expressed as log_10_ TCID_50_/0.2 mL; **f**, **g**); 3) histopathological changes in the lungs (**i**, **j**), on Days 3 (*n* = 3/group) and 7 (*n* = 3/group) after challenge. Efficacy against virus transmission in contact sentinel animals (*n* = 2/group) was evaluated by detection of viral load (**d**, **h**), lung pathology scoring (**i**, **j**). Control included an adjuvant-alone group. Scale bars are 500 µm. Differences in the studied parameters between animal groups were assessed using Tukey’s (virus titer; Lung Histology), Dunnett’s (postchallenge weight), or Šídák’s (viral load in PCR) multiple comparisons test. A *P* < 0.05 value was considered as a significant difference. * in comparison with the control group. LOD limit of detection.

On Day 21 after booster injection, immunized and control hamsters were intranasally challenged with 1×10^4^ TCID_50_ WT SARS-CoV-2 virus. Weights were monitored daily for 7 days after challenge infection (Fig. 5a). Only animals vaccinated with SWE-adjuvanted rSpike protein showed an increase in body weight during the period of observation, with all other groups losing weight. In animals that received SWE-adjuvanted rRBD vaccine, bodyweight stayed stable on day 2–3, but from Day 4 there was a reduction which reached a maximum (1.77% loss) on Day 7. In the control group, there was a steady decline in body weight reaching 9.8% reduction on Day 7. There was a significant difference in weight between the SWE-adjuvanted rSpike protein and the control group on Days 4–7 after challenge, and the SWE-adjuvanted rRBD and the control group on Day 7 post challenge. In addition to weight loss, some of the control animals showed clinical signs of infection with depressed activity and disheveled appearance, but similar signs were not seen in the vaccinated animals.

Viral load was determined in cell suspensions of nasal turbinates and lungs on Days 3 and 7 after challenge by real-time PCR (expressed in cycles/Ct) and by culture of infectious virus (log_10_ TCID_50_/0.2 mL). Viral RNA was positive by PCR in nasal turbinates and lungs on Day 3 after challenge in all groups (Fig. 5b, c). However, on Day 7 after challenge there was a complete absence of viral RNA (Ct > 40) in nasal turbinates and lungs only in the group that received SWE-adjuvanted rSpike protein (Fig. 5c). As detection of viral RNA by PCR early after challenge may just reflect the residual challenge virus, a more specific test for active infection is to measure culturable virus. Notably, animals vaccinated with SWE-adjuvanted rSpike protein had undetectable infectious virus in oropharyngeal swabs taken on Day 2 or nasal turbinate and lung samples on Days 3 and 7 post challenge (Fig. 5e, f, g). By contrast, infectious virus was detectable in Day 2 oropharyngeal swabs and Day 3 nasal turbinate and lung samples in all control animals, as well as animals vaccinated with SWE-adjuvanted rRBD vaccine although infectious virus levels in the latter were significantly lower than in the control group. On Day 7 after challenge, infectious virus was still detectable at low levels in nasal turbinate and lung samples of 2/3 animals in the control group but none of the vaccinated animals.

To test for an effect of vaccination on ability of a challenged animal to transmit virus, two naive sentinel animals were introduced into each cage containing infected animals on Day 2 after challenge. The sentinels was left in contact with the infected animals for one day and then removed and housed alone for a further 4 days before sacrifice. On Day 7 post challenge, all infected and sentinel animals were euthanized and viral load assessed in the nasal turbinates and lungs. Notably, only animals immunized with SWE-adjuvanted rSpike vaccine did not transmit infection to the contact sentinel animals, as indicated by the absence in their nasal turbinates or lung samples of viral RNA by PCR (Fig. 5d) or infectious virus by culture (Fig. 5h). By contrast, sentinels placed in the infected control group cage demonstrated evidence of active virus transmission, with viral RNA detected by PCR and infectious virus cultured at high titers from their nasal turbinates (3.62 ± 1.12) and lungs (1.5 ± 1.0 log_10_ TCID_50_/0.2 mL). The control sentinels demonstrated a 3.5 - 4.8% body weight decrease consistent with active infection. The sentinels placed in the SWE-adjuvanted rRBD group also showed evidence of virus transmission, with virus detectable in their nasal turbinates by both PCR and virus culture.

Next lung histology was assessed in infected animals to assess the extent of clinical disease. With the exception of the SWE-adjuvanted rSpike protein group, the lungs of all infected hamsters showed classic histological signs of acute respiratory distress syndrome (ARDS) caused by SARS-CoV-2 infection. Morphological characterization of the lungs in the SWE-adjuvanted rRBD and control groups demonstrated signs of the exudative phase of ARDS on Day 3 post-infection and fibroproliferative phase of ARDS on Day 7 post infection (Fig. 5j). Similar lung pathology was seen in the sentinel animals placed in contact with the infected SWE-adjuvanted rRBD and control groups. Scoring of lung pathology, confirmed the highest pathology scores in the control animals followed by the SWE-adjuvanted rRBD group who had significantly lower lung scores than the control group (Fig. 5i). Notably, only the SWE-adjuvanted rSpike protein group and their contact sentinel animals showed no signs of lung pathology, consistent with the absence of infectious lung virus on Day 3 and 7 post challenge in these animals, consistent with the prevention of virus transmission (Fig. 5j).

**Fig. 6:**
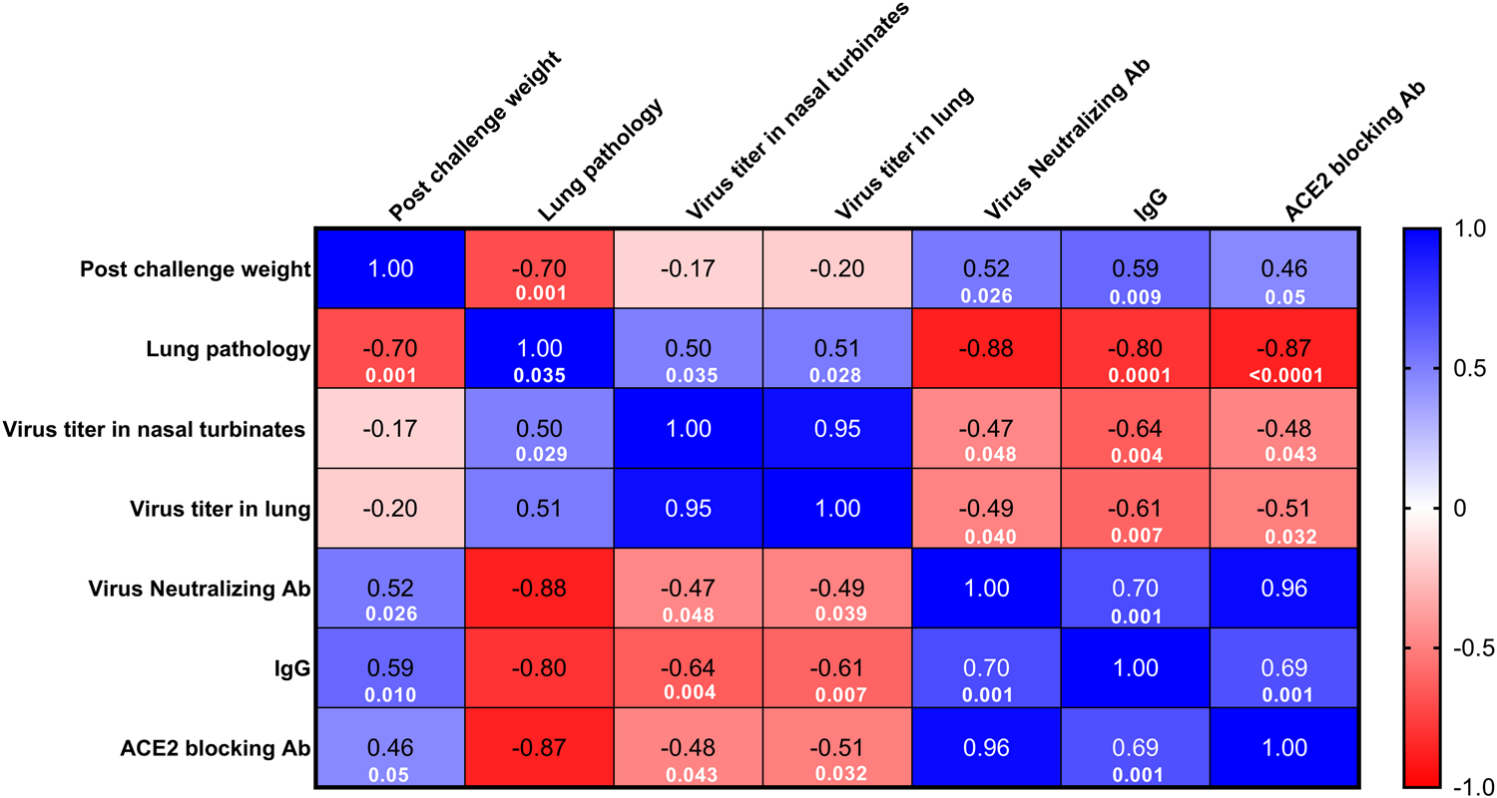
Correlation matrix analysis between immune markers including various antibody measurements and measures of vaccine protection including weight changes, viral loads, and lung pathology. The color refers to r value scale (−1 to 1) shown on the right. The number in each cell indicates the actual *r* value and the lower digits represent p-values. The analysis was conducted by multivariable Pearson correlation method.

Next, to identify the best correlate of protection, we evaluated the correlation between immune markers including various antibody measurements and measures of vaccine protection including weight changes, viral loads, and lung pathology (Fig. 6). The was a strong correlation between weight loss and lung pathology scores (*r* = 0.70, *P* = *0.001*), suggesting weight changes are a useful non-invasive measure for assessing disease severity. Notably, the strongest correlate of protection was found between prechallenge levels of anti-spike IgG, RBD-ACE2 binding inhibition and WT SARS-CoV-2 neutralizing antibodies and lung pathology scores, consistent with serum antibody levels post-vaccination being the best predictor of lung protection from infection.

## Discussion

Subunit vaccines are well-established as a reliable and safe platform effective against a variety of infectious diseases such as hepatitis B, diphtheria, pertussis, shingles and human papillomavirus^8^. An approved subunit vaccine against SARS-CoV-2 would represent an important step in the fight against the COVID-19 pandemic. Among the 110 COVID-19 vaccine candidates in clinical trials, the largest number (37, 34%) are subunit preparations^5^. Approximately half (16/37) of these candidates use full-length spike protein, with the rest focused on RBD (mostly its monomeric form^9^) or peptides^5,10^. Our research group consisting of a consortium of academic institutions including the International Center for Vaccinology at the Kazakh National Agrarian Research University (KazNARU) and the Aikimbayev National Research Center for Especially Dangerous Infections of the Ministry of Health of the Republic of Kazakhstan has been developing a protein subunit vaccine against COVID-19 called NARUVAX-C19 in collaboration with Australian biotechnology company, Vaxine Pty Ltd. The vaccine is prepared from the spike ECD expressed in insect cells using the baculovirus transfection system. The spike ECD has an amino acid sequence corresponding to wild-type SARS-CoV-2 (Wuhan strain) together with some stabilizing mutations. This spike ECD protein has been previously characterized in mice and ferrets as part of a vaccine called COVAX-19/SpikoGen^11^ that has progressed through phase III clinical trials and recently received emergency use authorization in Iran^5^. Adjuvants are generally needed to increase the effectiveness of subunit vaccines^12^. In the current studies, we combined this spike ECD protein with a squalene-containing oil-water emulsion type adjuvant SWE (Seppic, France), which is analogous to MF59, another well-known emulsion adjuvant. SWE is also being used as part of a subunit vaccine from the University of Saskatchewan which is in Phase I/II trials^5^. In our earlier study in cats^13^, we showed that SWE compared to aluminum hydroxide adjuvant provided better induction of IgG and neutralizing antibodies, as well as protection against SARS-CoV-2 infection.

We compared NARUVAX-C19 to another vaccine based on recombinant monomeric spike RBD protein, a popular target for COVID-19 vaccine development. Spike RBD is responsible for SARS-CoV-2 binding to the ACE-2 receptor for cell entry^14^ and RBD protein has been favored by some vaccine researchers because of its small size and easy expression at high concentrations in various expression systems^15^. However, RBD has fewer B and T cell epitopes than full-length spike protein^16^. Although neutralizing antibody is directed at the RBD, it is now recognized that neutralizing antibodies are also directed at other regions of the spike protein including the N-terminal domain and the fusion peptide, epitopes not present in RBD vaccines, which may explain why RBD-based vaccines are less protective than full spike protein vaccines as seen here.

In the mouse model, we showed the ability of SWE-adjuvanted rRBD and rSpike vaccines to elicit humoral and T-cell immune responses. SWE-adjuvanted rSpike protein, particularly the 2.5 µg dose, induced the highest levels of spike-binding IgG (IgG1 and IgG2a), RBD-ACE2 blocking antibodies and neutralizing antibodies against WT and delta variant of SARS-CoV-2 after both prime and booster immunizations. By comparison, even when formulated with the same SWE adjuvant, the rRBD vaccine even after two immunization failed to induce significant titers of virus-neutralizing antibodies in mice. The low immunogenicity of the RBD antigen is consistent with data from a phase I trial of a recombinant tandem-repeat dimeric RBD vaccine (ZF2001) which required 3 doses of 25 µg of the RBD antigen to ensure seroconversion of neutralizing antibodies in 97% of participants^17^. It is interesting that in our study the 2.5 µg dose induced higher levels of antibodies than the 5 µg dose. Indeed, similar observations of inverse spike protein dose effects on antibody production was seen in a previous ferret study using the same spike protein as used here^11^ and was also reported in the Novavax Phase I study where the lower 5 μg dose in the study was associated with higher antibody levels than the much higher 25 μg dose^18^. This suggests that excess doses of spike protein may in some way modulate and suppress the humoral immune response, thereby explaining why lower doses could result in high antibody responses.

The ideal vaccine should elicit a strong Th1 cellular response^19^ as this is important for SARS-CoV-2 protection^20^. In mice, our SWE-adjuvanted spike protein induced the strongest Th1 cellular immune response, as indicated by antigen-stimulated cytokine production and CD4 + and CD8 + T cell proliferation and IL-2 and TNF production. The Syrian hamster model is a sensitive model of SARS-CoV-2 infection^21^. In hamsters, the RBD vaccine was confirmed as a weak antigen that induced only low levels antigen-specific IgG and RBD-ACE2 blocking antibodies with minimal neutralizing antibody. This translated into only modest protection in response to challenge with heterologous SARS-CoV-2 D614G mutant^22^, with the RBD-immunized hamsters showing significant weight loss and detectable virus in Day 2 nasopharyngeal swabs and Day 3 nasal bulb and lung tissue. By contrast, the SWE-adjuvanted spike protein induced high levels of spike-binding IgG, RBD-ACE2 blocking antibodies and neutralizing antibody, including against the delta variant, that were higher than the antibody levels in convalescent infected hamster sera. The SWE-adjuvanted spike protein vaccine provided robust protection with the animals gaining rather than losing weight after challenge, and having no recoverable virus in Day 2 oropharyngeal swabs or Day 3 nasal bulb and lung samples. Only the animals immunized with SWE-adjuvanted spike protein did not transmit the infection to naïve sentinel mice placed into their cages Day 2 after challenge.

In studies in hamsters or humanized mice of adenoviral vector^23,24^ or mRNA^25^ vaccines, immunized animals after infection still demonstrated infectious virus in their upper respiratory tract. Similarly, non-human primates immunized with the AstraZeneca adenoviral vector vaccine post challenge showed equal amounts of infectious SARS-CoV-2 virus in the nasal bulb as unvaccinated animals^26^. Also, ferrets and hamsters immunized with a spike protein vaccine based on a HIV-trimerization domain and MF59 adjuvant showed similar levels of nasal virus as unvaccinated control animals^27^. Hence, these other COVID-19 vaccines do not appear to induce significant mucosal protection. Modest protection was observed after immunization with a subunit spike protein vaccine with a nitrogen bisphosphonate-modified zinc-aluminum hybrid adjuvant (FH002C) where after three doses of vaccine a 50% reduction in virus transmission to naive contact animals was observed^28^. The protection against transmission we observed in our study in the SWE-adjuvated rSpike protein group was consistent with the lack of detectable infectious virus in Day 2 oropharyngeal swabs and Day 3 nasal turbinates and lungs, normally the time where peak virus replication occurs in the hamster model^29^. Our SARS-CoV-2 virus TCID_50_ assay had an extremely low infective titer detection limit of 0.7 log_10_ TCID_50_/0.2 ml. Notably, the presence of residual virus RNA does not indicate the presence of infectious virus. We chose to use the TCID_50_ assay to detect virus rather than measure subgenomic RT-PCR^30,31^ as in our experience the TCID_50_ method is the most reliable to detect infectious virus. While we do not yet know the mechanism whereby our SWE-adjuvated rSpike protein suppressed nasal virus replication, this finding is extremely promising as it suggests potential for a transmission-blocking COVID-19 vaccine, something that has so far proved elusive.

There is an ongoing search for strong correlates of vaccine protection to help guide future vaccine approvals. We looked to see what parameters pre-challenge might best predict the challenge outcome. This analysis showed that serum neutralizing antibody titers pre-challenge had a high correlation with lung protection post challenge, consistent with the fact that COVID-19 vaccines appear most effective at protecting against serious disease and death^32^. While there was also a correlation between neutralizing antibody titers and Day 3 virus titers in the nasal bulb, this was not as strong which suggests that some other immune mediator such as memory T cells may play a role in mucosal protection seen with SWE-adjuvanted rSpike protein.

Our study has a number of limitations, as time and resources did not allow larger group sizes or the challenge and transmission study to be repeated. Nevertheless, the immunogenicity data obtained for the vaccines were consistent in mice and hamsters, in both cases rSpike protein showing potency greater over rRBD. Future planned studies will explore the ability of our SWE-adjuvanted rSpike protein vaccine to protect against challenge with the delta virus variant. This will be important as the delta strain has a high prevalence of globally, and there is increasing evidence that existing COVID-19 vaccines provide suboptimal protection against delta virus infections. Hamsters are currently the most used model for transmission studies^33^, although it is not known if such studies will predict human transmission. Hence, further studies are needed to define the mechanism whereby our spike protein vaccine was able to prevent nasal virus replication and transmission. In addition to future research to evaluate the protective efficacy in hamsters of NARUVAX-C19 vaccine against SARS-CoV-2 variants of concern including the delta variant, we also plan to evaluate protection by NARUVAX-C19 vaccine in the non-human primate model.

A subunit vaccine based on a recombinant spike protein extracellular domain was immunogenic in mice and provided robust protection of Syrian hamsters against infection by wild-type SARS-CoV-2 (D614G) virus in addition to blocking virus transmission to naïve contact animals. The full rSpike ECD antigen was significantly more effective than the antigen only comprising the spike rRBD. The protection with rSpike ECD correlated with high titers of neutralizing antibodies against the homologous wild-type virus that were also able to cross-neutralize the delta variant virus. Given this promising data, NARUVAX-C19 vaccine will now be evaluated in the non-human primate model, before advancing to human clinical trials.

## Methods

### Viruses and cell culture

The virus strain hCoV-19/Kazakhstan/KazNAU-NSCEDI481/2020 of wildtype SARS-CoV-2 with D614G mutation in spike protein was used. This virus was isolated at the Aikimbayev National Research Center for Especially Dangerous Infections (NSCEDI) in June 2020 from nasopharyngeal swab of a 45-year-old COVID-19 patient in Almaty, Kazakhstan (GISAID, #EPI_ISL_514093). We also used the delta variant (B.1.617.2) of SARS-CoV-2 isolated in August 17, 2021 from nasopharyngeal swab of a COVID-19 patient in Zhanaozen, Kazakhstan. Viruses were grown in Vero E6 cells (Vero 76, clone E6, CRL-1586, ATCC) with DMEM medium plus 2% fetal bovine serum (FBS) and antibiotic-antimycotic (containing penicillin 10,000 U, streptomycin 10 mg and amphotericin B 25 μg; #15240096, Gibco™) at 37 °C and 5% CO_2_ for 3 days. The challenge virus used was at the passage level 3 with an infectivity titer of 6.2–7.2 log_10_ TCID_50_/ml.

### Recombinant spike protein

The detailed methodology of obtaining spike protein has been described previously^8^. Briefly, the spike protein was identified from the SARS-CoV-2 genomic sequence in NCBI (access number: NC 045512)^34^. The codon-optimized insect cell expression cassette was cloned into pFASTBac1 and baculovirus was generated according to standard Bac-to-Bac procedures. The recombinant baculovirus was multiplied in Sf9 cells until the third passage and then used to infect High Five™ cells to express the protein. After 72 h of infection, the cell culture supernatant was purified by centrifugation and the recombinant ECD spike protein was purified on a HisTrap Excel column using an AKTA chromatography system, concentrated by ultrafiltration and replaced with PBS, sterilized by filtration. The sequence of the recombinant spike protein (rSpike) was confirmed by mass spectroscopy, SDS-PAGE gel, and Western blotting. Endotoxin was detected with the PyroGene™ Endotoxin Detection System (Cat. No. 50-658U, LONZA, Walkersville, MD, USA), and residual DNA content in the final vaccine product was determined with the Quant-iT™ PicoGreen™ dsDNA Assay Kit (ThermoFisher, P7589) according to manufacturers’ instructions.

### Vaccine formulation

Commercial spike rRBD protein was obtained from ABP Biosciences. This rRBD protein [Gln321-Ser591] was produced in HEK293 cells and had a stated purity > 95% as determined by SDS-PAGE and endotoxin < 1.0 EU per μg protein as determined by the LAL method.

Vaccine antigens (rSpike protein or rRBD) were formulated with Sepivac SWE™ adjuvant (SWE; Seppic, France) in a 50:50 ratio (by volume). SWE adjuvant with PBS was used as a negative control. All the preparations were sterile and contained less than 2.0 EU endotoxin per dose. After the final vaccine formulations were obtained, they were stored at 2–8 °C and used to immunize animals in the next day.

### Mice vaccination and immune response analysis

Specific pathogen-free BALB/c female mice 4–6-week-old were obtained from the NSCEDI’s breeding facility (Almaty, Kazakhstan). Animals were placed in ventilated cages with HEPA filters (Allentown, USA) for 7 days prior to the experiment for acclimatization. Mice (*n* = 7–10/group) were immunized intramuscularly (into the thigh area) in a volume of 100 μl twice at 21-day intervals. At 21 days after prime and booster vaccination, blood samples were collected from the orbital venous sinus and serum stored at -20˚C until used for antibody analysis (IgG *n* = 7–10/group; IgG1/IgG2a, RBD-ACE2 blocking and virus neutralizing Ab *n* = 7/group). At 21 days after prime and booster vaccination, mice (*n* = 4/group) were euthanized (cervical dislocation under ketamine/xylazine anesthesia) and their spleens collected under aseptic conditions to assess T-cell responses.

### Antibody analysis by ELISA

Ninety-six well microplates (Nunc MaxiSorp, #2297421, Invitrogen, USA) were coated with pretitrated 0.5 µg/ml rRBD or rSpike protein on commercial buffer (ELISA Coating Buffer, #B288159, BioLegend) overnight. Plates were blocked using ELISA Assay Diluent (#421203, BioLegend) 200 µl/well and incubated under constant shaking (300-330 rpm on a PST-60HL thermal shaker, BIOSAN) for 1 h at room temperature. The plates were washed four times with ELISA Wash Buffer (# 421601, BioLegend). Mouse serum samples were titrated two-fold from dilutions 1:125 to 1:32,000 (after prime vaccination) or 1:512000 (after booster vaccination), 100 µl samples were added from each dilution to the wells and incubated under constant shaking (300-330 rpm) for 1.5-2 h at room temperature. After washing (4x), secondary anti-mouse biotinylated IgG detection antibody (1:4000, #B304057, BioLegend), IgG1, IgG2a (1:1000, #B270354, B268020, BioLegend, 100 µl/well) was added and the plates were incubated (1 h at room temperature with shaking). After additional washing (4x), plates were incubated with HRP Streptavidin (# 405210, BioLegend, 1:1000, 100 µl/well) for 30 min at room temperature with shaking. Finally, plates were washed (5x) and added ready-to-use TMB substrate (#N301, Thermo Fisher Scientific, 100 µl/well). The color reaction was stopped by adding a stop solution (#B308260, BioLegend, 100 µl/well), and the optical density was measured (measuring wavelength 450 nm, reference wavelength 630 nm) on a Stat Fax 2100 analyzer (Awareness Tech). The cut-off value for determining the titer was calculated based on the average optical density (OD) values of the wells containing only the buffer (blank) + three standard deviation.

### Determination of RBD-ACE2 binding inhibition

The SARS-CoV-2 Surrogate Virus Neutralization Test (sVNT) Kit (L00847; GenScript, Piscataway, USA) was used according to the manufacturer’s instructions. Briefly, samples and controls were incubated with HRP-conjugated RBD (HRP-RBD) at 37 °C for 30 min. Mixtures were added to a hACE2-coated capture plate and incubated at 37 °C for 15 min. The plates were then washed to remove the HRP-RBD neutralizing antibody complexes and allowing the unbound HRP-RBD and HRP-RBD non-neutralizing antibody complexes to bind to hACE2. TMB solution was added and incubated at room temperature for 15 min, and the reaction was stopped with a stop solution. The OD was measured by spectrophotometry at 450 nm. The percentage of inhibition of the sample was calculated as (1-Average OD of the sample/Average OD of the negative control) × 100%. A sample with an inhibition percentage <30% was considered “negative” and ≥30% “positive” for RBD-ACE2 binding antibodies. The following RBD-ACE2 binding antibody values were determined according to the level of inhibition: low (30–59%), medium (60–89%), high (≥90).

### Virus neutralizing antibody analysis in mice samples

Serum samples were complement inactivated at 56 °C for 30 min then serially two-fold diluted in medium (DMEM-2% FCS-1% Antibiotic-Antimycotic; in final dilutions of 1:20 to 1:2560) and incubated in duplicates at 1:1 ratio with 1000 TCID_50_ of SARS-CoV-2 D614G mutant. After 1 h incubation at room temperature, the serum-virus mixture was transferred to a 96-well plate (#3596, Corning) covered with a monolayer (obtained by seeding with 5 ×10^4^ cells per well and 24 h incubation, the confluence was about 95%) of Vero-E6 cell culture. After 1 h, the inoculums were removed, fresh medium was added, and the plates were incubated at 37 °C and 5% CO_2_ for 3 days. The neutralizing antibody titer was the highest dilution of serum that inhibited the cytopathic effect in 100% of wells. The cytopathic effect was assessed visually using a MIB-R trinocular inverted biological microscope (LOMO-Microsystems, Russia) with a magnification of x10.

### Analysis of cytokines

Mice spleens were mechanically pulverized into single-cell suspension using a cell strainer (Falcon® 70 µm). The pulverization procedure was performed on a disposable sterile Petri dish (#840007, Piove di Sacco, Italy) with 10 ml of 3% fetal bovine serum (FBS, US Origin, #VP1810311, Millipore Corp., Germany) in PBS. Erythrocytes were lysed with RBC lysis buffer (#B333276, BioLegend). Splenocytes were cultured in a 5% CO_2_ incubator (INCO 153, Memmert, Germany) at 37 °C in 24-well flat-bottomed plates (#04618024, Sigma-Aldrich, USA) at 1 × 10^6^ cells/well (1 ml) in RPMI-1640 + GlutaMax medium (#2242279, Gibco) with 20 mM HEPES (#15630-080, Gibco), 10% FBS (inactivated by heating) and 1% Antibiotic-Antimycotic (#15240096, Gibco™) in the presence of 5 µg of recombinant RBD/Spike protein or without protein (control without stimulation). Concanavalin A (#11028-71-0, InvivoGen) was used as a positive control or T-cell mitogen at a concentration of 50 µg/ml. Cells were incubated for 72 h, after which the supernatant was examined for cytokines IL-2 (#B320273), IFN-γ (#B307222), IL-4 (#B320413), IL-10 (#B311304), IL-5 (#B317463), IL-6 (#B321215), IL-17A (#B303513), TNF-α (#B306271) using ELISA MAX™ Deluxe Set Mouse (BioLegend) kits, according to manufacturer instructions. The data were presented as the difference (Delta) in cytokine concentrations between samples with and without vaccine protein stimulation, which are presented in pg/mL.

### Analysis of CD4 + and CD8 + T cell proliferation

T cell proliferation assay was performed by incubating isolated splenocytes for 5 min in the dark on ice with 5 µM CFSE (Carboxyfluorescein succinimidyl ester, eBioscienceTM, #2298273, Invitrogen, USA), and immediately the staining was quenched with 3% FBS. Cells were cultured at 10^6^ cells/ml in 24-well plates for 5 days at 37 °C in 5% CO_2_ with or without 5 µg/ml of rRBD or rSpike protein. Phenotype of cells was determined by analysis of CD markers-stained cells using Flow Cytometry. Briefly, cells were first incubated with TruStain FcX™ (anti-mouse CD16/32, to block nonspecific binding of immunoglobulin to Fc receptors) at a concentration of 0.5 µg/10^6^ cells for 5-10 min on ice before immunostaining. The cells were then incubated with antibodies specific to surface markers for 30 min in the dark on ice. The following fluorochrome-labeled antibodies were used: R-PE anti-CD8a (#2170194, Invitrogen, USA) at a concentration of 0.25 µg, violetFluorTM 450-anti-CD4 (#ab241097, Abcam) at a concentration of 0.125 µg per 100 µl of splenocyte suspension. After incubation, cells were resuspended in Flow Cytometry Staining Buffer (eBioscience, #2231157, Invitrogen, USA) up to 0.5 ml, stained with 0.25 µg of 7-AAD solution, and incubated for 5-10 min in the dark (BioLegend Cat. No. 420403) to exclude dead cells. At least 5×10^5^ cells were analyzed for each sample on an Attune™ NxT flow cytometer (Thermo Fisher Scientific, USA) using Attune NxT Software (Thermo Fisher Scientific, USA). T-cell population was analyzed in the lymphocyte gate isolated on FCS/SSC dot-plot. CD4 + and CD8 + T cell proliferation was calculated as the difference (Δ) in antigen-stimulated and unstimulated samples to the total number of live proliferating (CFSE + ) lymphocytes and expressed as a percentage (Supplementary Figure S1).

### Vaccination of Syrian hamsters and collection of blood samples

Six- to eight-week-old male Syrian hamsters obtained from the NSCEDI’s laboratory animal breeding facility were used. Animals were placed in ventilated cages with HEPA filters (Allentown, USA) for 7 days prior to the experiment for acclimatization. Hamsters were immunized with rRBD and rSpike-based vaccine formulations containing 5 µg/dose antigen and a control sample intramuscularly (into the thigh area) in a volume of 200 µl twice at 21-day intervals. On day 21 after booster (*n* = 6/group) vaccination, blood samples were collected from the hyoid venous plexus (under ketamine/xylazine anesthesia) and serum was stored at -20˚C until used in antibody analysis. For determining the virus-neutralizing antibodies 1000 TCID_50_ of wild-type (D614G) or delta variant of SARS-CoV-2 were used.

### Antibody analysis in hamster samples

ELISA was performed as described above for the mouse samples. Except the hamster serum samples were titrated starting from dilution 1:125 to 1:256000. Secondary Goat Anti-Syrian Hamster IgG H&L biotinylated antibody (1:4000, #ab6891, Abcam, MA, USA) was used for IgG antibody detection. Determination of RBD-ACE2 blocking antibodies and virus-neutralizing antibody titers were determined against SARS-CoV-2 wild-type with D614G mutant and delta variant viruses as described above. Serum samples from four hamsters previously infected with the wild-type SARS-CoV-2 virus with a D614G mutation in the spike protein were used for comparison. Serum samples from previously infected hamsters were taken 21 days after the challenge.

### Assessing the vaccine protective efficacy in hamsters

On day 21 after booster injection, hamsters were infected with WT SARS-CoV-2 at a dose of 1 × 10^4^ TCID_50_ intranasally under intraperitoneal ketamine (100 mg/kg) and xylazine (10 mg/kg) anesthesia. Virus material was diluted in DMEM medium and injected 100 µl into the nose (approximately 50 µl in each nostril) using a pipette. Animals were observed for 7 days twice daily after challenge and body weight recorded daily. On Days 3 and 7 after infection, half of the animals (3/6) from each group were euthanized and collected nasal turbinate and lung samples. Three lobes of the right lung from each animal were fixed in 10% formalin for histopathological examination. Two lobes of the left lung were homogenized in 1 ml DMEM using a TissueLyser II instrument (QIAGEN) at 300 vibrations/min for 60 s, supernatant collected after centrifugation (5000 g for 15 min at 4 °C) was stored at -70 °C for detection of viral RNA and infectious viral titer.

### Virus transmission analysis

In all the vaccinated groups of hamsters, on the second day after challenge, two naive SARS-CoV-2 hamsters were introduced (per three vaccinated animals one sentinel in the cage with dimensions: 19” X 10” X 8”). Before co-housing the sentinels with infected animals, oropharyngeal swabs collected from all the challenged hamsters on Day 2 after infection to assess for virus load. The sentinels were left in contact with the infected animals for just one day and then removed and housed alone for a further 4 days before sacrifice. Both the sentinel animals were then euthanized to assess viral load in the nasal turbinates and lungs by RT-PCR and virus culture, and their lungs were evaluated by histology.

### Real-time reverse transcriptional polymer-chain reaction

Quantification of viral RNA in the samples was performed using a one-step Real-time RT-PCR. Tissue homogenate samples were used to isolate viral RNA using the QIAamp Viral RNA Mini Kit (Qiagen, Hilden, Germany) according to the manufacturer’s instructions. RNA was eluted in 50 μl of elution buffer and used for detection with a commercial real-time RT-PCR kit (NSCEDI) according to the manufacturer’s instructions. The following primer pairs targeting to the N gene of the SARS-CoV-2 virus were used: F 5′ - GGGGAACTTCTCCTGCTAGAAT; R 5′ - CAGACATTTTGCTCTCAAGCTG. Amplification was performed as follows: 50 °C for 10 min, 95˚C for 2 min, then 45 cycles consisting of 95 °C for 15 s, 60 °C for 30 s and a default melting curve in the RotorGene® machine (QIAGEN, USA). When the Ct values (cycles) on the FAM/Green and JOE/Yellow channels were ≥40, the samples were considered negative for SARS-CoV-2.

### Detection of infectious titer load

Live virus titers in the respiratory tract tissue homogenates were determined by 50% Tissue Culture Infectious Dose assay (TCID_50_). The tissue homogenates were diluted 10-fold in medium (DMEM-2% FCS-1% Antibiotic-Antimycotic) and transferred in quadruplicates to 96-well plates containing confluent Vero-E6 cells, incubated at 37 °C and 5% CO_2_ for 5 days. Titration results were counted visually by microscopic examination of the cell monolayer for characteristic cytopathogenic effects (rounding and detachment of cells from the monolayer). Virus titer was calculated according to the method of Reed and Mench and expressed in log_10_ TCID_50_/0.2 mL.

### Histological analysis of hamster lungs

Hamster lungs were fixed in 10% neutral buffer formaldehyde after excision, washed in water and then subjected to treatment with 4 portions of isopropyl 100% alcohol and 2 portions of xylene. The material was then impregnated in four portions of paraffin and blocks were casted. The histological blocks were sectioned (5 μm thick) using a microprocessor-controlled microtome MZP-01 (KB Technom, Russia). The sections were dewaxed in 2 portions of xylene and 3 portions of ethyl alcohol with decreasing concentration (96°, 80°, 70°), and then stained with Hematoxylin (#05-002, BioVitrum, Russia)—Eosin (#C0362, DiaPath, Italy). This was followed by clarification in ascending ethyl alcohols (70°, 80°, 96°) and two portions of xylene. The sections were covered with coverslips using Bio Mount synthetic medium (#2813, Bio Optica, Italy). The preparations were studied using an Mshot microscope (China), model MF52-N. Photographs were taken at x40 magnification using an Mshot MS23 camera attachment (China) in the MShot Image Analysis System program (China). A magnification of x1000 was achieved with an oil immersion lens, and a standardized scale was used for calibration. All the measurements were made in μm. Lung microscopic examination was performed according to classical canons accepted for parenchymatous organs, and the narrative was constructed according to the description of pathological conditions caused by SARS-CoV and SARS-CoV-2^35,36^. Each slide was quantified based on the severity of histologic changes, including interstitial pneumonia, alveolitis, bronchiolitis, alveolar destruction, interstitial infiltration, pulmonary hemorrhage, and peribronchiolar inflammation. Scoring of pathological changes in the lung sections: 4 points = extremely severe; 3 points = severe; 2 points = moderate; 1 point = mild; 0 point = no changes^37^.

### Biosafety and bioethics

All the work with the SARS-CoV-2 and animal experiments with the virus were conducted in NSCEDI’s BSL-3 and ABSL-3 laboratories, wherein the international standard ISO 35001:2019 “Biorisk management for laboratories and other related organizations” was followed. Laboratory animals were kept in individually ventilated cages (Tecniplast, Italy & Allentown, US) under a 12/12 light regime. The present study was conducted in accordance with national and international laws and guidelines for the handling of laboratory animals. The protocol was approved by the Institutional Committee on the Keeping and Use of Laboratory Animals of the NSCEI, Protocol No. 4 dated September 22, 2020.

### Statistical analysis

The GraphPad Prism version 9.0.0 software program (San Diego, CA, USA) was used for the statistical analysis of data. Differences in antibody titers, cytokine production, T-cell proliferation, weight loss, viral load, and pathological changes in the lungs between animal groups were assessed using Tukey’s multiple comparison test, Šídák’s multiple comparison test or Dunnett’s multiple comparison test. Statistical analysis of IgG, IgG1, IgG2a, and neutralizing antibody titers was done using log_2_ transformed data. The detection limit of the virus titer was 0.7 log_10_ TCID_50_/0.2 mL. The detection limit of IgG titers and its isotypes was 7.0 log_2_; neutralizing antibodies 3.0 log_2_. Geometric mean titers (GMT) with 95% confidence interval were calculated for all types of antibody data. The effect of different types of antibodies on vaccine protectiveness indicators such as weight dynamics, viral load in respiratory organs, and the level of pathological changes in the lungs was assessed using the multivariable Pearson correlation method. For all comparisons, *P* < 0.05 was considered statically significant.

### Reporting summary

Further information on research design is available in the Nature Research Reporting Summary linked to this article.

## Supplementary information

REPORTING SUMMARY

Figure S1

## Data Availability

Data are available from the corresponding author upon request.
