# Supplementary material for: An adjuvanted subunit SARS-CoV-2 spike protein vaccine provides protection against Covid-19 infection and transmission
Source: NPJ Vaccines. 2022 Feb 23;7:24. doi: 10.1038/s41541-022-00450-8 (PMC8866462; doi:10.1038/s41541-022-00450-8)
Supplement: Supplementary file 1 — REPORTING SUMMARY [file 41541_2022_450_MOESM1_ESM.pdf]

nature portfolio

Corresponding author(s): Prof. Tabynov, NPJVACCINES-01692R

Last updated by author(s): Nov 19, 2021

Reporting Summary

Nature Portfolio wishes to improve the reproducibility of the work that we publish. This form provides structure for consistency and transparency in reporting. For further information on Nature Portfolio policies, see our Editorial Policies and the Editorial Policy Checklist.

Statistics

For all statistical analyses, confirm that the following items are present in the figure legend, table legend, main text, or Methods section.

n/a	Confirmed	
☐	☒	The exact sample size (*n*) for each experimental group/condition, given as a discrete number and unit of measurement
☒	☐	A statement on whether measurements were taken from distinct samples or whether the same sample was measured repeatedly
☐	☒	The statistical test(s) used AND whether they are one- or two-sided<br>*Only common tests should be described solely by name; describe more complex techniques in the Methods section.*
☒	☐	A description of all covariates tested
☒	☐	A description of any assumptions or corrections, such as tests of normality and adjustment for multiple comparisons
☒	☐	A full description of the statistical parameters including central tendency (e.g. means) or other basic estimates (e.g. regression coefficient) AND variation (e.g. standard deviation) or associated estimates of uncertainty (e.g. confidence intervals)
☒	☐	For null hypothesis testing, the test statistic (e.g. *F*, *t*, *r*) with confidence intervals, effect sizes, degrees of freedom and *P* value noted<br>*Give P values as exact values whenever suitable.*
☒	☐	For Bayesian analysis, information on the choice of priors and Markov chain Monte Carlo settings
☒	☐	For hierarchical and complex designs, identification of the appropriate level for tests and full reporting of outcomes
☒	☐	Estimates of effect sizes (e.g. Cohen's *d*, Pearson's *r*), indicating how they were calculated

Our web collection on statistics for biologists contains articles on many of the points above.

Software and code

Policy information about availability of computer code

Data collection	*Provide a description of all commercial, open source and custom code used to collect the data in this study, specifying the version used OR state that no software was used.*
Data analysis	The GraphPad Prism version 9.0.0 software program (San Diego, CA, USA) was used for statistical analysis of data

For manuscripts utilizing custom algorithms or software that are central to the research but not yet described in published literature, software must be made available to editors and reviewers. We strongly encourage code deposition in a community repository (e.g. GitHub). See the Nature Portfolio guidelines for submitting code & software for further information.

Data

Policy information about availability of data

All manuscripts must include a data availability statement. This statement should provide the following information, where applicable:

- Accession codes, unique identifiers, or web links for publicly available datasets
- A description of any restrictions on data availability
- For clinical datasets or third party data, please ensure that the statement adheres to our policy

All data supporting the experimental findings of this study are available within the manuscript and are available from the corresponding author upon request

Field-specific reporting

Please select the one below that is the best fit for your research. If you are not sure, read the appropriate sections before making your selection.

☒ Life sciences ☐ Behavioural & social sciences ☐ Ecological, evolutionary & environmental sciences

For a reference copy of the document with all sections, see nature.com/documents/nr-reporting-summary-flat.pdf

Life sciences study design

All studies must disclose on these points even when the disclosure is negative.

Sample size	Sample sizes were based on similar experimental designs by others in the field contemporaneously.
Data exclusions	None
Replication	No
Randomization	Animals were randomized into groups by weight
Blinding	The study was not blinded to group data. Blinding is uncommonly done in small animal experiments such as these.

Reporting for specific materials, systems and methods

We require information from authors about some types of materials, experimental systems and methods used in many studies. Here, indicate whether each material, system or method listed is relevant to your study. If you are not sure if a list item applies to your research, read the appropriate section before selecting a response.

Materials & experimental systems

n/a	Involved in the study	
☐	☒	Antibodies
☐	☒	Eukaryotic cell lines
☒	☐	Palaeontology and archaeology
☐	☒	Animals and other organisms
☒	☐	Human research participants
☒	☐	Clinical data
☒	☐	Dual use research of concern

Methods

n/a	Involved in the study	
☒	☐	ChIP-seq
☐	☒	Flow cytometry
☒	☐	MRI-based neuroimaging

Antibodies

Antibodies used	Anti-mouse biotinylated IgG detection antibody (#B304057, BioLegend), IgG1, IgG2a (#B270354, B268020, BioLegend); TruStain FcX™ (anti-mouse CD16/32); R-PE anti-CD8a (#2170194, Invitrogen, USA); violetFluorTM 450-anti-CD4 (#ab241097, Abcam)
Validation	Validation done by the use of controls as mentioned in the MS

Eukaryotic cell lines

Policy information about cell lines

Cell line source(s)	Vero E6 cells (Vero 76, clone E6, CRL-1586, ATCC); High Five™ cells (ThermoFisher Scientific)
Authentication	Cell lines were not authenticated
Mycoplasma contamination	Vero E6 cells were certified Mycoplasma sp free. This was subsequently tested by PCR testing.
Commonly misidentified lines (See ICLAC register)	None

Animals and other organisms

Policy information about studies involving animals; ARRIVE guidelines recommended for reporting animal research

Laboratory animals	Specific pathogen-free BALB/c female mice 4-6-week-old (n=85) were obtained from the NSCEDI's breeding facility (Almaty, Kazakhstan).<br>Six- to eight-week-old male Syrian hamsters (n=24) obtained from the NSCEDI's laboratory animal breeding facility were used.
Wild animals	N/A
Field-collected samples	N/A
Ethics oversight	Laboratory animals were kept in individually ventilated cages (Tecniplast, Italy & Allentown, US) under a 12/12 light regime. The present study was conducted in accordance with national and international laws and guidelines for handling of laboratory animals. The protocol was approved by the Institutional Committee on the Keeping and Use of Laboratory Animals of the NSCEI, Protocol No. 4 dated September 22, 2020.

Note that full information on the approval of the study protocol must also be provided in the manuscript.

Flow Cytometry

Plots

Confirm that:

☒ The axis labels state the marker and fluorochrome used (e.g. CD4-FITC).

☐ The axis scales are clearly visible. Include numbers along axes only for bottom left plot of group (a 'group' is an analysis of identical markers).

☐ All plots are contour plots with outliers or pseudocolor plots.

☒ A numerical value for number of cells or percentage (with statistics) is provided.

Methodology

Sample preparation	T cell proliferation assay was performed by incubating isolated splenocytes for 5 min in the dark on ice with 5 µM CFSE (Carboxyfluorescein succinimidyl ester, eBioscienceTM, #2298273, Invitrogen, USA), and immediately the staining was quenched with 3% FBS. Cells were cultured at 106 cells/ml in 24-well plates for 5 days at 37°C in 5% CO2 with or without 5 µg/ml of RBD or Spike protein. Phenotype of cells was determined by analysis of CD markers-stained cells using Flow Cytometry. Briefly, cells were first incubated with TruStain FcX™ (anti-mouse CD16/32, to block nonspecific binding of immunoglobulin to Fc receptors) at a concentration of 0.5 µg/106 cells for 5-10 min on ice before immunostaining. The cells were then incubated with antibodies specific to surface markers for 30 min in the dark on ice. The following fluorochrome-labeled antibodies were used: R-PE anti-CD8a (#2170194, Invitrogen, USA) at a concentration of 0.25 µg, violetFluorTM 450-anti-CD4 (#ab241097, Abcam) at a concentration of 0.125 µg per 100 µl of splenocyte suspension. After incubation, cells were resuspended in Flow Cytometry Staining Buffer (eBioscience, #2231157, Invitrogen, USA) up to 0.5 ml, stained with 0.25 µg of 7-AAD solution, and incubated for 5-10 minutes in the dark (BioLegend Cat. No. 420403) to exclude dead cells.
Instrument	Attune™ NxT flow cytometer (Thermo Fisher Scientific, USA)
Software	Attune NxT Software (Thermo Fisher Scientific, USA)
Cell population abundance	At least 5x105 cells were analyzed for each sample.
Gating strategy	T-cell population was analyzed in the lymphocyte gate isolated on FCS/SSC dot-plot. CD4+ and CD8+ T cell proliferation was calculated as the difference (Δ) in antigen stimulated and unstimulated samples to the total number of live proliferating (CFSE+) splenocytes and expressed as a percentage.

☐ Tick this box to confirm that a figure exemplifying the gating strategy is provided in the Supplementary Information.
