# Supplementary material for: An adjuvanted subunit SARS-CoV-2 spike protein vaccine provides protection against Covid-19 infection and transmission
Source: NPJ Vaccines. 2022 Feb 23;7:24. doi: 10.1038/s41541-022-00450-8 (PMC8866462; doi:10.1038/s41541-022-00450-8)
Supplement: Supplementary file 2 — Figure S1 [file 41541_2022_450_MOESM2_ESM.pdf]

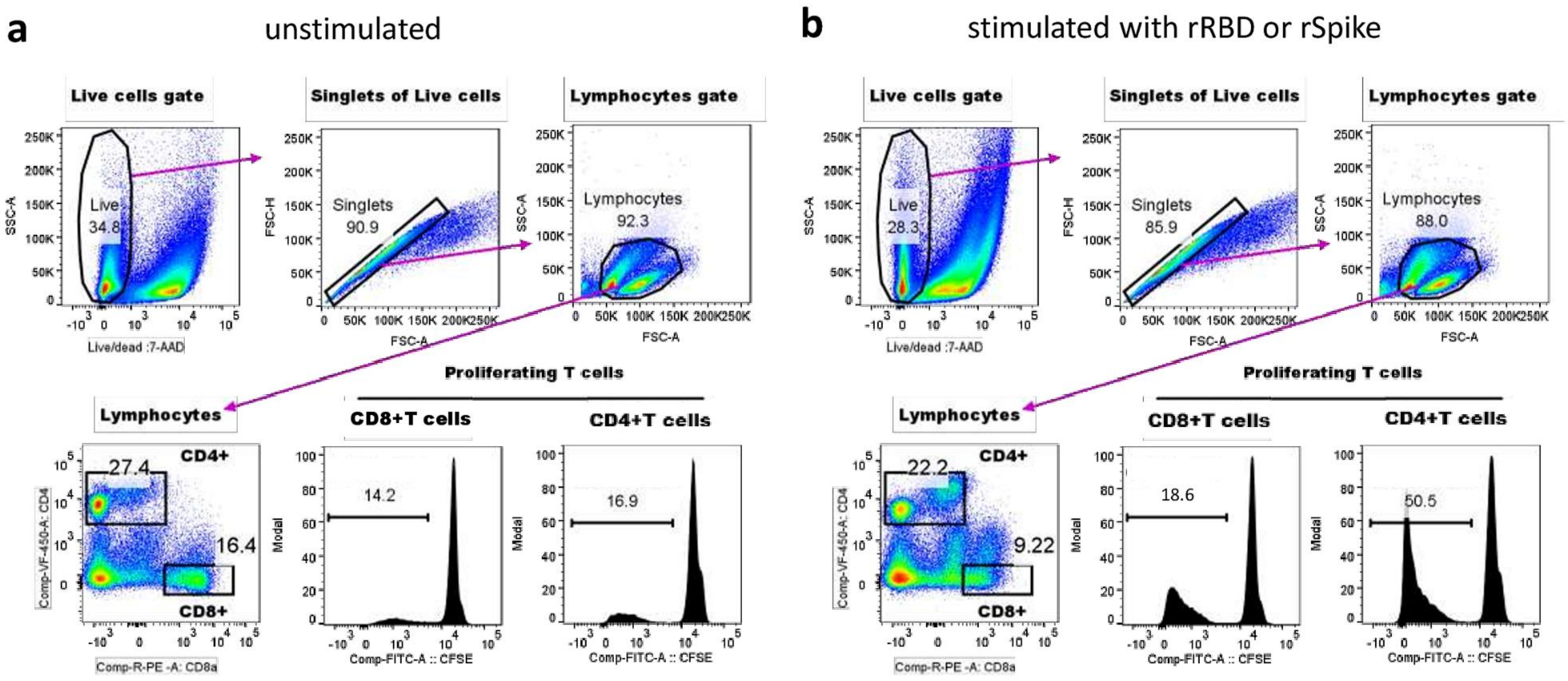

Figure S1: Gating strategy used to study CD4+ and CD8+ T cell proliferation by CFSE decay. T-cell population was analyzed in the lymphocyte gate isolated on FCS/SSC dot-plot. Doublets (FSC-H and FSC-A gating), dead cells (fixable viability stain) were excluded from the analysis. CD4+ and CD8+ T cell proliferation was calculated as the difference (Δ) in antigen unstimulated (**a**) and stimulated samples (**b**) to the total number of live proliferating
